# Mass Cytometry Analysis Reveals Complex Cell-State Modifications of Blood Myeloid Cells During HIV Infection

**DOI:** 10.3389/fimmu.2019.02677

**Published:** 2019-11-22

**Authors:** Sixtine Coindre, Nicolas Tchitchek, Lamine Alaoui, Bruno Vaslin, Christine Bourgeois, Cecile Goujard, Camille Lecuroux, Pierre Bruhns, Roger Le Grand, Anne-Sophie Beignon, Olivier Lambotte, Benoit Favier

**Affiliations:** ^1^CEA-Université Paris Sud-INSERM U1184, IDMIT Department, IBFJ, DRF, Fontenay-aux-Roses, France; ^2^Service de médecine interne et d'immunologie clinique, Hôpital Bicêtre, APHP, Le Kremlin Bicêtre, France; ^3^INSERM U1018-Université Paris Sud, CESP (Centre for Research in Epidemiology and Population Health), Le Kremlin Bicêtre, France; ^4^Unit of Antibodies in Therapy and Pathology, Institut Pasteur, UMR1222 INSERM, Paris, France

**Keywords:** LILRB1 (ILT2), LILRB2 (ILT4), LILRA4 (ILT7), CD32 (FcgRII), CD38, immune checkpoints, primary HIV infection, elite controllers

## Abstract

Dendritic cells (DC), which are involved in orchestrating early immune responses against pathogens, are dysregulated in their function by HIV infection. This dysregulation likely contributes to tip the balance toward viral persistence. Different DC subpopulations, including classical (cDCs) and plasmacytoid (pDCs) dendritic cells, are subjected to concomitant inflammatory and immunoregulatory events during HIV infection, which hampers the precise characterization of their regulation through classical approaches. Here, we carried out mass cytometry analysis of blood samples from early HIV-infected patients that were longitudinally collected before and after 1 year of effective combination antiretroviral therapy (cART). Blood samples from HIV controller patients who naturally control the infection were also included. Our data revealed that plasma HIV RNA level was positively associated with a loss of cDC and pDC subpopulations that display high expression of LILR immunomodulatory receptors. Conversely, specific monocyte populations co-expressing high levels of HLA-I, 3 immunomodulatory receptors, CD64, LILRA2, and LILRB4, and the restriction factor CD317 (also known as BST2/Tetherin), were more abundant in early HIV-infection. Finally, our analysis revealed that the blood of HIV controller patients contained in a higher abundance a particular subtype of CD1c^+^ cDCs, characterized by elevated co-expression of CD32b inhibitory receptor and HLA-DR antigen-presentation molecules. Overall, this study unravels the modifications induced in DC and monocyte subpopulations in different HIV^+^ conditions, and provides a better comprehension of the immune regulation/dysregulation mechanisms induced during this viral infection.

## Introduction

HIV infection is characterized by the dysregulation of immune responses leading to viral persistence and disease progression (1–3). During the last few decades, most studies of HIV pathogenesis have focused on T-cell immune responses. Nevertheless, dendritic cells (DCs) including, classical (cDCs) and plasmacytoid (pDCs) cells, play a pivotal role in the early defenses against viruses by bridging innate and adaptive immune responses (4, 5). After viral sensing, cDCs rapidly mature and migrate toward secondary lymphoid organs to stimulate T-cell responses. Several studies indicate that in HIV or SIV infections, cDCs are prone to apoptosis and demonstrate attenuated capacities of antigen presentation and cytokine production leading to inefficient T-cell proliferation (1, 6–8). *Ex vivo* analysis of cDCs from HIV-infected patients illustrates phenotypic changes induced early during infection and that are associated with cDC dysregulation (9, 10). Further studies in rhesus macaques identify dysregulation of cDCs induced in early SIV infection as a predictive marker of disease progression (11). These studies suggest a critical role for cDCs in the regulation of early immune responses, where deficiencies in functions tip the balance of disease outcomes toward viral persistence.

Because pDCs show unique capacities to regulate immune responses and viral replication through massive production of type I interferon (IFN), their role in HIV and SIV infection has also been investigated. pDCs from chronically HIV-infected patients show dysregulated immunophenotypic attributes (12). *In vitro* experiments indicate that HIV attenuates the production of type I-IFNs mediated by pDCs (13). Moreover, during early SIV infection, pDCs rapidly move toward lymph nodes, are subjected to apoptosis and renewal, and only a small fraction of these cells produce type-I-IFNs (14, 15). These data suggest that SIV infection induces heterogeneous functional capacities among pDCs.

Massive monocyte turnover is induced during SIV and HIV infection and has been directly linked to disease progression (3, 14). In addition, microbial translocation induces overactivation of monocytes, which in turn participate in the inflammatory events associated with viral persistence (3, 15). Finally, the production of soluble CD14 and CD163, which reflects monocyte/macrophage activation, has been associated with HIV mortality in primary and chronic infection (3, 15–17).

Even though these studies indicate that DC and monocyte subpopulations are dysregulated in HIV infection, a precise view of their dysregulation mechanisms at the molecular level is difficult to decipher through classical approaches. In this respect, HIV infection induces concomitant inflammatory and immunoregulatory events, which can differentially influence cell maturation/activation phenotype within the same populations due to proximity and/or exposure to different stimuli (virus and host mediators). Phenotypic heterogeneity among subpopulations may be further enhanced by perturbation of hematopoiesis and egress of less differentiated DCs from bone marrow to replenish dying cells as has been explored in SIV infection (18, 19).

In this study, we carried out a mass cytometry analysis to unravel the heterogeneity and dynamics of myeloid cell subsets occurring from the acute phase of HIV infection to the control of viral replication through successful combination antiretroviral therapy (cART). For this purpose, we collected samples from primary HIV-infected patients longitudinally, prior to and after 1 year of effective cART. Samples from elite controllers, who naturally control HIV replication in the absence of treatment, were also included as well as control samples from healthy donors. Interestingly, myeloid cells from elite controllers were previously shown to display enhanced functions and a specific expression profile of Leukocyte Immunoglobulin-Like Receptors (LILRs), a family of receptors that play important roles in the regulation of myeloid cell maturation and functions (20–22). In this regard, LILRs could represent key markers that account for DC-associated regulation/dysregulation mechanisms (23). Therefore, markers for the most well-characterized LILRs were included in our mass cytometry panel.

Our data reveal an association between a high level of HIV RNA and a loss in the blood of cDC and pDC clusters that highly expressed specific members of LILR family. In contrast, early HIV infection was positively associated with clusters of monocytes displaying high expression of HLA-I ligands, CD64, LILRA2, LILRB4 immunoregulatory receptors and restriction factor CD317 (also known as BST2 or tetherin), a ligand of LILRA4. Finally, a subtype of cDCs defined by high expression of CD32b and HLA-DR was more abundant in elite controllers than in other conditions. Altogether, our results provide a unique view of the diversity and various phenotypic changes induced in DCs and monocytes during early HIV infection, before and after effective cART, but also in patients that naturally control HIV infection. Overall, this study reveals new insights on the mechanisms driving the dysregulation of early myeloid immune responses, which may account for inefficient adaptive immune responses and viral persistence.

## Results

### Phenotypical Characterization of Dendritic Cell and Monocyte Populations Among Patient Samples

To characterize the phenotypic diversity of DC and monocyte subsets in HIV primary and controlled infections, we developed a mass-cytometry panel of 29 markers mainly dedicated to myeloid cells (Table S1). We applied this panel to PBMCs from three groups of individuals. The first group included six patients who were longitudinally sampled during the primary phase of HIV infection, as previously described (24), before (primary HIV) and after 1 year of combination antiretroviral therapy (HIV cART). The second group was composed of six HIV-infected elite controller patients (HIV controllers) that naturally control viral replication. Finally, the third group encompassed six healthy subjects (Healthy). Clinical characteristics of these groups are shown in Table 1. These groups allow us to question the changes in the dynamics, diversification, and regulatory events among DCs and monocytes during both primary infection and potential normalization on cART, and in HIV controllers.

**Table 1:** Summary of patient and subject clinical parameters.

	**Primary HIV (*n* = 6)**	**HIV cART (*n* = 6)**	**HIV controllers (*n* = 6)**	**Healthy (*n* = 6)**
**Age** median (min-max), in years	34 (24–47)	35 (25–48)	39 (25–49)	35 (25–45)
**Gender**	M	M	M	M
**Days since HIV-1 diagnosis** median (min-max), in days	28 (18–29)	361 (290–372)	1,825 (1,825–7,300)	N/A
**Treatment**	Naive	cART	Naive	Naive
**RNA HIV load at diagnosis** median (min-max), in log_10_ copies/ml of plasma	6.67 (5.47–7.26)	1 (1–1.63)	1.54 (1.43–1.69)	N/A
**CD4 T-cell count** median (min-max), in 10^3^ cells/μl of blood	470 (258–669)	843 (570–1,247)	827 (652–1,180)	856 (634–1,412)

*Eighteen Caucasian men were involved in this study to constitute four groups of conditions. These groups are composed of six primary HIV-infected patients before treatment (primary HIV) and after 1 year of combination antiretroviral therapy (HIV cART), six elite controllers (HIV controllers), and six healthy subjects (Healthy). The gender and median patient age, days since HIV-1 diagnosis, RNA viral load, and CD4^+^ T-cell count are indicated for each group. cART, combination antiretroviral therapy; N/A, not applicable; M, male*.

**Figure 1:**
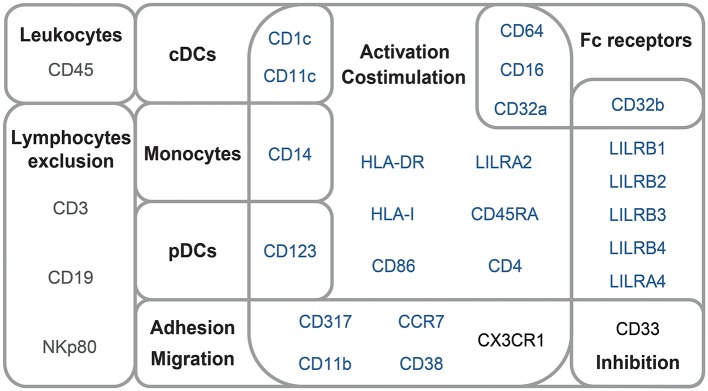
Mass-cytometry panel. Mass-cytometry panel of 29 cell parameters used to characterize myeloid cell population diversity in PBMC samples. Markers used to select leukocytes and exclude lymphocytes from the analysis are indicated in gray. Markers used for SPADE clustering of myeloid cells are indicated in blue. Markers used for the phenotypic characterization of myeloid cells are indicated in blue or black.

**Figure 2:**
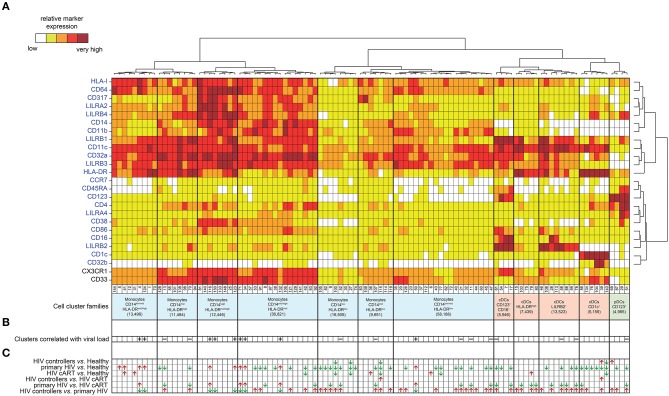
Phenotypic landscape, variation in cell abundance and association with HIV-infection of myeloid cell clusters from HIV-infected and healthy donor samples. **(A)** Heatmap showing relative marker expression for myeloid cell clusters. The mean of the median expression of each marker was determined and classified using a five-tiered color scale ranging from white (not expressed) to dark red (highly expressed), according to their relative ranges of expression (5th−95th percentile) throughout the dataset. Clustering markers are shown in blue. Hierarchical clustering of both the cell clusters and clustering markers were performed and represented using dendrograms. Based on the cluster dendrogram, several cluster families were identified and represented in blue for monocyte families, red for cDC families and green for pDC family. **(B)** Chart summarizing clusters having cell abundance correlated with HIV RNA load (Correlated Clusters). Clusters positively correlated are indicated with a “+,” whereas clusters negatively correlated are indicated with a “–.” **(C)** Chart summarizing the clusters showing significantly differences of cell abundances between the biological conditions (Differentially Abundant Clusters). For each identified DACs, red arrows indicate an increase of the cell cluster abundance, whereas green arrows indicate a decrease of the cell cluster abundance.

The mass cytometry panel was designed to detect lineage, migration and adhesion markers, as well as activation and inhibitory immunoreceptors known to play an important role in myeloid cell functions and maturation (Figure 1). After the acquisition of all samples by mass-cytometry, we applied a first step of manual gating strategy to exclude lymphocyte subsets, followed by the positive selection of cell populations expressing HLA-DR (Figure S1) to select DCs and other myeloid cells. We next characterized the simultaneous expression of markers from our panel on these myeloid cells. For this purpose, we used the Spanning-tree Progression Analysis of Density-normalized Events (SPADE) clustering algorithm to identify myeloid cell clusters having similar expressions for selected markers regardless of their sample cell origin (25, 26). A categorical heatmap was generated using hierarchical clustering to visualize more easily the respective relative marker expression of each myeloid cell cluster identified (Figure 2A). The overall phenotype distribution of all clusters was visualized in Figure S2. For each cluster, the correlation with plasma RNA viral load and its variation in abundance across patient groups was also analyzed (Figures 2B,C).

Based on the cell cluster dendrogram, 12 families of cell clusters were defined (Figure 2A). A tSNE representation generated to represent the similarities between cell cluster phenotypes confirmed the segregation of cell cluster families defined by the heatmap dendrogram (Figure S3). Seven cluster families exhibited a monocyte phenotype (HLA-DR^+^, CD14^+^) and 4 cluster families exhibited a cDC phenotype (CD14^−^, CD11c^+^, HLADR^+^). Among the 4 cluster families with a cDC phenotype, one family highly expressed CD123, CD86, and CD16. Finally, a family of cell clusters corresponding to the phenotype of pDC (CD14^−^, CD11c^low^, CD123^+^, CD4^+^, HLADR^+^) was defined. A total of 76 cell clusters were associated with the monocyte families, whereas cDC and pDC families consisted of 23 and 4 cell clusters, respectively. Cell abundance of each cluster was represented in Figure S4.

These results indicate that monocytes and DCs in the dataset include heterogeneous and discrete cell subpopulations carrying a specific combination of markers that may reflect a divergence in functions or differentiation that could be associated with HIV infection.

### Primary HIV-Infection Induces a Significant Loss of Peripheral cDC2 and pDCs

**Figure 3:**
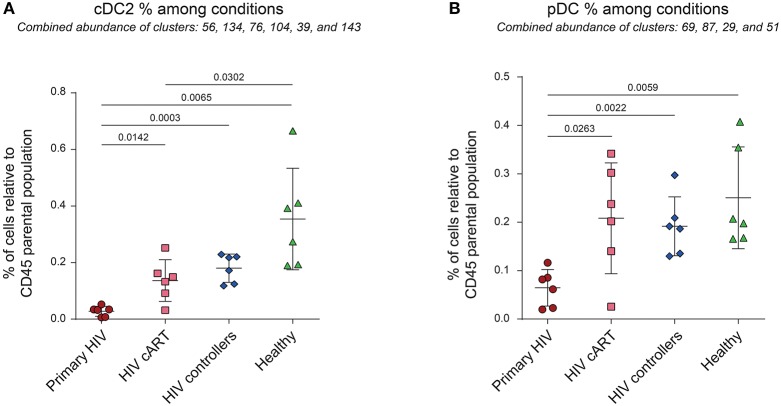
Percentages of cDC2 and pDC among CD45^+^ cells from HIV-infected patients and healthy donors. **(A,B)** Percentage analyses of cDC2 (left panel) and pDC (right panel) relative to CD45^+^ cells among conditions. Percentages were compared among primary HIV-infected patients before (Primary HIV, red circles) and after 1 year of cART (HIV cART, pink squares), HIV-infected elite controller patients (HIV controllers, blue rhombus), and healthy donors (Healthy, green triangles). Statistical differences between conditions were calculated using a two-tailed unpaired Welch's *t*-test with a *p*-value threshold of 0.05.

Previous reports show the loss of DC subpopulations during early HIV and SIV infection (10, 11). To assess this process in our dataset, we first analyzed the variations of cell cluster abundances for the cDC2 (CD1c^+^ cDC) and pDC families among the different conditions. The percentage of cells in each condition relative to the number of cells in the CD45^+^ parent population were compared (Figure 3). We observed significant decreases of the percentages of cDC2 (80% of decline, *p* = 0.0142) and pDC (69% of decline, *p* = 0.00263) in the blood of primary HIV-infected patients compared to the same patients 1 year after effective cART. These significant decreases were also observed for primary HIV-infected patients in comparison to HIV controllers (85% of cDC2 decline, *p* = 0.0003 and 66% of pDC decline, *p* = 0.0022) and to healthy donors (92% of cDC2 decline, *p* = 0.0065 and 74% of pDC decline, *p* = 0.0059). Interestingly, our results also demonstrated significantly higher percentages of cDC2 (*p* = 0.0302) in the healthy group than in the HIV cART-treated group (Figure 3A). No significant difference was observed between these two groups for pDCs (Figure 3B). Concordantly with previous studies, our dataset demonstrates that primary HIV-infection induces a profound decline of peripheral cDC2s and pDCs abundance that seems to be partially recovered under cART, but to a lesser extent for cDC2s.

### Elevated Plasma HIV Load Is Associated With Modifications in the Abundance of Specific Dendritic Cell and Monocyte Subpopulations

We next investigated if the abundance of specific myeloid cell subsets was associated with HIV RNA levels across HIV^+^ and healthy samples.

**Figure 4:**
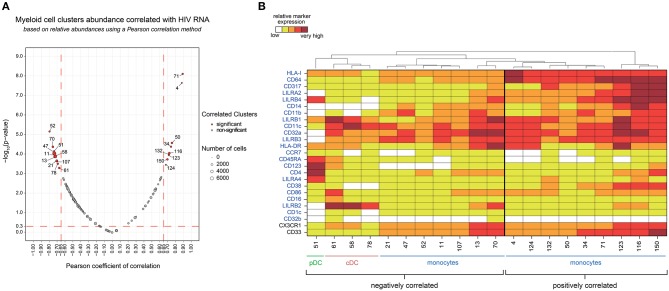
Association analysis of myeloid cell cluster abundances with HIV RNA levels and phenotypic characterization. **(A)** Two-dimensional chart representing the correlation between myeloid cell clusters abundances and the total HIV RNA. Correlations were identified based on the number of cells associated with each cluster relative to the number of cells in the CD45^+^ parent population. Each dot in the representation corresponds to a cell cluster. The size of the dot is proportional to the number of cells of the whole dataset associated with the cluster. Significantly positively (right) or negatively (left) correlated clusters are indicated in red with a Pearson correlation coefficient >0.65 and a *p* < 0.05. The Pearson correlation coefficient is represented on the X-axis and the associated *p*-value, shown as −log_10_, on the Y-axis. **(B)** Heatmap representation showing the phenotype of the clusters positively and negatively correlated with HIV RNA levels. The relative marker expression for each cluster was indicated by a five-tiered color scale ranging from white (not expressed) to dark red (highly expressed). Clustering markers are indicated in blue. Using cluster dendrogram and CD14, CD11c, and CD123 expression level, clusters were annotated as monocyte, cDC or pDC.

We found 20 clusters significantly correlated (CCs) with plasma viral load (Figure 4A). These clusters were split into two groups based on the positive or negative correlation of their cell abundance with HIV RNA load (Figure 4B). On the one hand, nine clusters that were positively correlated with viral load were exclusively monocyte populations. On the other hand, eleven clusters that negatively correlated with viral load included monocyte and DC populations. The monocyte clusters that positively correlated with viral load were mainly characterized by strong expression of CD64 and HLA-I. These clusters also displayed high expression of HIV restriction factor CD317, and the immunomodulatory receptors LILRA2 and LILRB4. It is important to note that among them, only some clusters, displayed a medium or high-level expression of CD38 (a transmembrane glycoprotein involved in myeloid cell adhesion, activation, and metabolism). Among the dendritic cell clusters that negatively correlated with the viral load, pDC cluster #51 (*R* = −0.65) demonstrated a strong expression of LILRA4 and LILRB4. Furthermore, cDC clusters #61 (*R* = −0.59), #58 (*R* = −0.66), and #78 (*R* = −0.60) expressed high levels of LILRB2 inhibitory receptor. Meanwhile, the seven monocyte clusters that negatively correlated with viral load expressed moderated levels of HLA-I, CD64, CD317, LILRA2, LILRB4, and CD38, in contrast to the other monocyte clusters that were positively correlated with HIV RNA. Interestingly, the negatively correlated monocyte clusters #13 and #70 displayed a medium and high expression of LILRB2, respectively, with high coexpression of CX3CR1 and CD33.

Our data show that an increase in HIV viral load was associated with the specific loss of discrete DC populations in blood. These populations included pDC clusters that were characterized by high expression of LILRA4 and LILRB4, but also cDCs highly expressing LILRB2. Conversely, specific subsets of monocytes strongly expressing HLA-I, CD64, CD317, LILRA2, LILRB4, and for some of them, CD38, seemed to be enriched when viral load increased.

### Primary HIV-Infected Patients and HIV Controllers Display Differentially Abundant Clusters

We then aimed to identify myeloid cell clusters undergoing contraction or expansion within the different patient groups. These clusters were named Differentially Abundant Clusters (DACs) and were summarized in Figure 2C. We first identified clusters having different cell abundances between healthy and each HIV^+^ conditions (Figures S5A–C). We found that DACs were mainly enriched in healthy condition. This may indicate expression level modification of surface markers or a loss in the blood of specific myeloid cell subsets in HIV-infected patients even under cART or natural control, compared to healthy donors. We then identified clusters with differences in cell abundances for the comparisons between all HIV^+^ groups (Figures S5D–F). The comparison between HIV controllers and primary HIV samples displayed the greatest number of DACs, with 43 out of 52 being more abundant in samples from HIV controllers (Figure S5F). Conversely, the comparison between samples from HIV controllers and HIV-infected patients once under cART displayed the lowest number of DACs, with only 2 DACs more abundant in HIV controllers (Figure S5D). These changes may indicate important differences in myeloid cell dynamics between primary HIV^+^ and HIV-controller patients, whereas the myeloid cell dynamics between cART treated HIV^+^ and HIV-controller patients is more similar.

**Figure 5:**
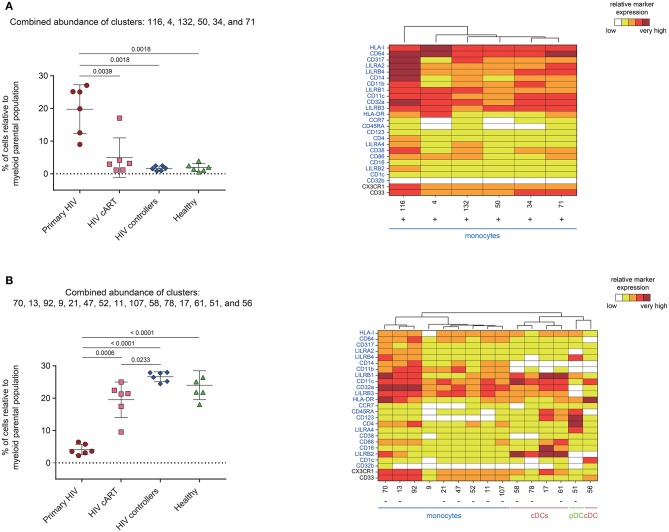
Characterization of myeloid cell clusters showing significant differences in cell abundance for primary HIV condition. **(A)** Graph showing the cell abundance of the clusters #116, #4, #132, #50, #34, and #71 relative to myeloid cells for all samples, and heatmap representation illustrating the phenotype of these clusters. These six clusters were significantly more abundant under primary HIV (red circles) compared to HIV cART (pink squares), HIV controllers (blue rhombus), and Healthy (green triangles) conditions. **(B)** Graph showing the cell abundance of the clusters #70, #13, #92, #9, #21, #47, #52, #11, #107, #58, #78, #17, #61, #51, and #56 relative to myeloid cells for all samples, and heatmap representation illustrating the phenotype of these clusters. These 15 clusters were significantly less abundant under primary HIV (red circles) compared to HIV cART (pink squares), HIV controllers (blue rhombus), and Healthy (green triangles) conditions. These clusters were also significantly less abundant under HIV cART condition compared to HIV controllers condition. In the abundance graph representations, for each condition, the mean cell abundance is indicated (black lines). Statistical differences between conditions were calculated using a two-tailed unpaired Welch's *t*-test with a *p*-value threshold of 0.05. For heatmap representations, the relative marker expression for each cluster was indicated by a five-tiered color scale ranging from white (not expressed) to dark red (highly expressed). Clustering markers are indicated in blue. Using cluster dendrogram and CD14, CD11c, and CD123 expression level, clusters were annotated as monocyte, cDC or pDC. Clusters positively correlated with HIV RNA levels are indicated with a “+” while clusters negatively correlated are indicated with a “–”.

We next analyzed the phenotype and abundance of all DACs that were significantly more abundant in primary HIV^+^ patients when compared to the other conditions (HIV cART, HIV controllers, and Healthy; Figure 5A). We found that six DACs, clusters #116, #4, #132, #50, #34, and #71, were more abundant in primary HIV^+^ samples and displayed monocyte phenotypes. Interestingly, these six clusters were positively correlated with plasma viral load (Figure 4). These results confirmed that individuals in the primary HIV infection state had a higher abundance of monocytes that strongly expressed HLA-I, CD64, CD317, LILRA2, and LILRB4, with some subpopulations also expressing mid- to high- levels of CD38.

We then focused on DACs that were less abundant in primary HIV^+^ patients than in all other conditions (Figure 5B). These clusters included: monocyte clusters #70, #13, #21, #47, #52, #11, and #107; cDC clusters #58, #78, and #61; and pDC cluster #51. These 11 clusters were previously identified as negatively correlated with viral load. The four remaining DACs less abundant in primary HIV^+^ samples displayed monocyte phenotypes (clusters #92 and #9), and cDC phenotypes (clusters #17, and #56). Furthermore, cDC clusters #58, #78, #17, and #61 strongly expressed LILRB2 immunoreceptor, with clusters #17 and #61 also expressing CD123, CD86, and CD16. Cluster #56 did not express LILRB2, but strongly expressed CD1c. The pDC cluster #51, which was also found correlated with HIV RNA in Figure 4, strongly expressed LILRA4 and LILRB4. It is interesting to note that all the DACs that were less abundant in primary HIV condition had a weaker expression of HLA-I, CD64, CD317, LILRA2, and LILRB4 compared to clusters more abundant in the same condition (Figure 5A). In addition, none of these DACs expressed a medium or high-level of CD38.

**Figure 6:**
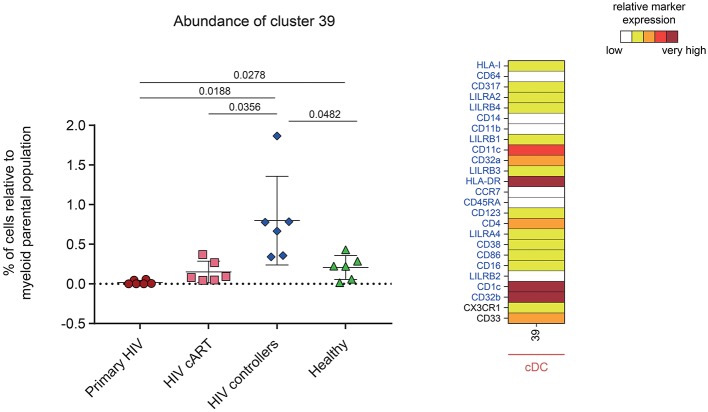
Characterization of a myeloid cell cluster specifically and significantly more abundant in HIV controller patients. Graph showing the cell abundance of the cluster #39 relative to myeloid cells for all samples, and heatmap representation illustrating the phenotype of this cluster. Cluster #39 was significantly more abundant in HIV controllers (blue rhombus) compared to primary HIV (red circles), HIV cART (pink squares), and Healthy (green triangles) conditions. This cluster was also significantly more abundant in Healthy donors compared to primary HIV infected patients. In the abundance graph representations, for each condition, the mean cell abundance is indicated (black lines). Statistical differences between conditions were calculated using a two-tailed unpaired Welch's *t*-test with a *p*-value threshold of 0.05. For heatmap representation, the relative marker expression for cluster #39 was indicated by a five-tiered color scale ranging from white (not expressed) to dark red (highly expressed). Clustering markers are indicated in blue. Using cluster CD14, CD11c, and CD123 expression level, cluster #39 was annotated as cDC.

One DAC was shown to be more abundant in HIV controllers compared to other HIV^+^ and healthy conditions (Figure 6). This DAC, cluster #39, displayed a CD1c^+^ cDC phenotype strongly expressing inhibitory receptor CD32b (low-affinity receptor for IgG also known as FcγRIIB) and HLA-DR. Furthermore, this cluster expressed moderate levels of CD32a (activatory counterpart of CD32b), CD4 and CD33. Using a Kolmogorov–Smirnov distance (KS) test to quantify the differences in marker expression between this cluster and all CD1c^+^ cDC subsets (Figure S6), we identified CD32b as the marker with the highest difference (KS = 0.7672). In addition, when we quantified the differences in marker expression between this cluster and the other CD32b^+^ CD1c^+^ cDC clusters (Figure S7), CD32b was the second most distant marker (KS = 0.5021). Thus, high CD32b expression was specific to this CD1c^+^ cDC subset found enriched in the blood of HIV controllers.

Altogether, we found that a modification in the balance of monocyte and DC subpopulations is induced from the early stages of HIV infection. Monocytes with a strong expression of HLA-I, CD64, CD317, LILRA2, LILRB4, and CD38 were most prominent in primary HIV^+^ patients when compared to cART, HIV controllers and healthy donors. In addition, primary HIV^+^ patients had significantly fewer pDCs that expressed high levels of LILRA4 and LILRB4. Moreover, they had considerably less LILRB2^hi^ cDCs. Finally, we discovered a unique CD1c^+^ CD32b^hi^ HLA-DR^hi^ cDC cluster that was specifically enriched in HIV elite controllers.

## Discussion

DCs and monocytes play an important role in the initiation of immune responses against HIV. However, the study of their regulation and dynamics during early HIV infection is hindered by late disease diagnosis. Moreover, early immune responses against HIV induce complex concomitant inflammatory and immunoregulatory events that can be difficult to decipher through classical approaches. In this regard, we carried out a mass cytometry analysis to characterize phenotypic heterogeneity among myeloid cell subpopulations under various conditions of HIV infection.

Our high dimensional analysis illustrates that specific DC and monocyte subpopulations found in peripheral blood are differentially affected by HIV infection. After mapping the different phenotypes of DC and monocyte subsets from PBMC samples, we highlighted a global loss of cDC2s and pDCs under primary HIV-infection in the blood. No DC clusters were enriched during primary infection, suggesting that this decrease could result from cell death rather than phenotypic changes (7, 10, 11). However, it is also well-established that cDCs and pDCs rapidly migrate toward peripheral lymph nodes after HIV or SIV infection to elicit adaptive immune response (27, 28). Therefore, the loss of DC subsets observed in primary HIV infection might result from various physiological mechanisms.

We also investigated the abundance variation of myeloid cell clusters among the different conditions and determined their association with HIV RNA level. We found that specific monocyte clusters were enriched in primary HIV-infected patients and/or positively correlated with the plasma viral load. These subpopulations of monocytes expressed high levels of HLA-I and CD64. In the context of viral infection, HLA-I molecules present peptides derived from intracellular viral proteins to CD8^+^ T lymphocytes to activate their cytolytic activity (29). CD64 is a high-affinity Fc receptor for IgG, which allows for immune complex internalization driving cross-presentation of viral epitopes on HLA-I (30). Thus, it seems consistent that monocytes positively correlated with the viral load exhibit strong co-expression of HLA-I and CD64. Increased HLA-I and CD64 expressions by monocytes were previously reported in acute and chronic HIV-infection, respectively (8, 31). However, our data demonstrate further the strong expression of CD317, LILRA2, and LILRB4 for these subsets, and for some subclusters, a medium or high-level expression of CD38. In agreement with our data, the HIV-restriction factor CD317 was previously shown to be up-regulated at the surface of monocytes and CD4^+^ T-cells during acute HIV/SIV infection (32, 33). The activating immune-receptor LILRA2 is expressed by monocytes and neutrophils and recognizes bacterially cleaved immunoglobulin, leading to the activation of signaling pathways and subsequent immune responses (34). However, LILRA2 can selectively modulate LPS-mediated cytokine production by monocytes, and could inhibit CD64-dependent phagocytosis (35). Finally, increased expression of LILRB4 was shown to induce tolerogenic monocytes and cDCs (8, 36–38). Previous studies have also demonstrated that LILRB4 interaction with CD64 is a potent inhibitor of monocyte activation, and CD64-mediated clathrin-dependent endocytosis and phagocytosis (39, 40). Thus, expression of these molecules could explain the dysregulated ability of phagocytosis and cytokine production observed in monocytes from HIV^+^ patients (41). The up-regulation of inhibitory receptors on immune cells could also constitute a retro control mechanism to dampen chronic inflammation.

Conversely, monocyte clusters that were negatively correlated with viral load, and/or less abundant in primary HIV-infected patients, did not exhibit high level expression of HLA-I, CD64, CD317, LILRA2, LILRB4, and CD38. Therefore, these monocyte subpopulations seemed to be specifically differentiated and/or contracted under primary HIV infection. Our mass cytometry panel included CD16 marker to detect inflammatory monocytes. However, we only detected few clusters of CD16^hi^ cDCs and no CD16^hi^ monocyte clusters. The attenuation of CD16 staining on monocytes could result from the freezing procedure of PBMCs that was shown to modify staining of some FcRs on specific cell subsets (42).

Among the DC populations characterized in our study, the clusters of cDCs that highly expressed LILRB2, and a cluster of pDCs that highly expressed LILRA4 and LILRB4, were also less abundant in primary HIV-infected patients. The majority of these clusters were also negatively correlated with plasma viral load. Functional studies indicate that cDCs and pDCs show dysregulated functions in early and chronic HIV/SIV infections. cDCs have an impaired maturation with reduced capacity for antigen presentation and cytokine production, whereas pDCs have reduced capacities to produce type-I IFNs (1, 9, 13, 43–45). In this respect, HIV infection enhances LILRB2/HLA-I and LILRA4/CD317 axes leading to the dysregulation of cDC and pDC functions, respectively (8, 9, 13, 46–49). Moreover, it has been reported that the strength of the LILRB2/HLA-I interaction is enhanced by HLA-I presentation of HIV-derived peptides, or genetic variation of HLA-I haplotypes, and correlates with the level of cDC dysregulation in HIV-infected patients (46, 47). Previous studies showed that LILRB2 was up-regulated on cDCs from blood in the acute phase of HIV and SIV infection (9, 10). LILRB2 expression was even higher in cDCs from peripheral lymph nodes than those from peripheral blood during early SIV infection (10). Therefore, in our study, the loss of LILRB2^hi^ cDC and LILRA4^hi^ pDC clusters observed in the blood from primary HIV infected patients could correspond in part to the migration of these subpopulations toward secondary lymphoid organs. Consequently, inhibitory impulses induced by the LILRB2/HLA-I and LILRA4/CD317 axes on cDCs and pDCs that have migrated into secondary lymphoid organs during primary HIV infection may enhance their dysfunctions and impaired the establishment of an effective adaptive immune response.

We also found that HIV controllers had an enrichment of a CD1c^+^ CD32b^hi^ HLA-DR^hi^ cDC cluster. These data are concordant with the recent discovery of a new CD1c^hi^, CD32b^hi^ cDC subset, using a single-cell RNA sequencing approach (50). This cDC subset was a potent stimulator of naive T-cell proliferation, and was more likely to secrete high levels of CCL19, IL-8, IL-10, and IL-12b (50). However, further studies will be necessary to better characterize the function of this DC subset in the context of HIV infection, in particular its implication in the mechanisms leading to the natural control of viral replication in elite controller patients. Since natural control of HIV infection can occur through various mechanisms, it will be important to also characterize the abundance of CD1c^+^ CD32b^hi^ HLA-DR^hi^ cDCs in additional elite controller patients, to assess whether this feature is shared by all elite controllers.

New populations of cDCs, called pre-DCs, were recently identified in the peripheral blood from healthy donors (51). These populations can differentiate into cDC1 or cDC2 subsets *in vitro* and were proposed to be cDC precursors. However, the relationship between pre-DCs and HIV infection is still unknown. Therefore, future mass cytometry studies including additional dendritic cell markers such as CD141, CD2, Siglec-6, and Axl will be helpful to better understand the dynamics and regulation of cDC1 and pre-DC subsets during HIV infection.

In conclusion, our results unravel the diversity and various phenotypic changes induced in cDCs, pDCs, and monocytes during early stage, anti-retroviral treatment control, and naturally controlled HIV infections. These results should be helpful when trying to better understand the cellular and molecular basis of the events driving the regulation/dysregulation of myeloid immune responses involved in the progression or control of HIV infection.

## Materials and Methods

### Study Subjects and Ethics Statements

This study involved six patients with primary HIV-1 infection estimated, as previously described (17), to have been infected <30 days before inclusion. These patients were enrolled from the French ANRS CO6 PRIMO cohort and has approval by the Ethics Committee of Cochin Hospital. Blood samples were collected from patients at enrolment, who were antiretroviral naïve and negative for hepatitis B and C viruses. The six primary HIV-infected patients were then placed under cART, and blood samples were collected after 1 year. As previously described for these individuals (24), after 1 year of cART viral load was undetectable and CD4^+^ T cell count was restored (CD4^+^ T cells > 500/μl of blood). This study also involved blood samples of six HIV controller patients from the French ANRS CO21 CODEX. This cohort was approved by the ethics review committee of Ile de France VII. HIV controllers were defined as patients infected by HIV-1 for at least 5 years, who never received cART and whose last five consecutive plasma HIV RNA values were <400 copies/ml. Finally, blood samples of six healthy subjects were obtained from the Etablissement Français du Sang (EFS). All subjects gave written informed consent to participate in the study.

### Sample Processing and Storage

As previously described (24), peripheral blood mononuclear cells (PBMCs) were isolated by Ficoll density gradient centrifugation, and ~1 × 10^7^ PBMCs per sample were cryopreserved at −150°C.

### Antibody Labeling and Cell Staining

To avoid batch variation effects, all samples were stained with the same batch of antibodies and on the same day. All samples were acquired in 1 day to avoid instrument signal fluctuation. Antibodies (listed in Table S1) were either pre-conjugated from the manufacturer (Fluidigm, San Francisco, CA) or conjugated in-house with the appropriate metal isotopes as previously described (24). Cells were thawed and 5.10^6^ PBMCs were transferred per well. As previously described (24), PBMCs were incubated with Rhodium DNA-intercalator (Fluidigm), first stained with the primary surface antibody mix for 1 h, and then stained with the secondary surface antibody mix for 15 min. Next, samples were resuspended in 1.6% PFA and incubated 20 min. Cells were finally incubated 30 min in permeabilization buffer with 1 μM Iridium DNA-intercalator before a 4°C overnight incubation in 1.6% PFA with 0.1 μM Iridium DNA intercalator. For acquisition, cells were washed and filtered through a cell strainer cap of a 5-ml polystyrene round-bottom tube (BD Biosciences). Normalization beads (Fluidigm) were added to each sample. Then, samples were acquired using a mass cytometer (CyTOF-I; Fluidigm) and following the standard procedure recommended by the manufacturer. An average of 200,264 ± 13,472 events was acquired per sample.

### Data Normalization

Raw cytometry profiles generated by CyTOF-I were normalized using normalization beads and MatLab Compiler normalizer software (52).

### Manual Gating of CD45^+^ Myeloid Cells

Normalized events were gated using the Cytobank analysis platform. First, cells with only one Iridium level were selected to exclude cell doublets from the analysis. Second, normalization beads and dead cells were removed by selecting cells negative for Cerium (Ce140) and Rhodium, respectively. Thirdly, leukocytes were selected based on the positive expression of CD45. Myeloid cells were then selected based on the absence of CD3, CD19, and NKp80, and expression of HLA-DR (Figure S1).

### Automatic Identification of Cell Clusters

SPADE algorithm was used to perform automatic identification of cell clusters (25). We observed large differences in the number of manually gated myeloid cells in each sample, ranging from 2,255 to 35,658 cells. Consequently, we first down-sampled 2,255 cells from each sample to avoid over-representation of samples with high number of cells. These uniformly down-sampled events were up-sampled at the end of the SPADE analysis. The SPADE clusters were generated using the entire and singular dataset (24 samples from 18 individuals, including 6 during primary infection and 1 year later after effective cART). Cells having similar expression of the 23 selected clustering markers (shown in blue in Figure 1) were grouped into clusters, regardless of donor origin. Moreover, SPADE was configured to identify 150 cell clusters (down-sampling of 40%). These parameters were defined to obtain the highest number of clusters with uniform phenotypes (26). The SPADE heatmap was generated with the SPADE clusters using the 23 clustering markers. CD45, CD3, CD19, and NKp80 were discarded from the SPADE analysis as we used them to pre-select cells of interest. Both CX3CR1 and CD33 markers were not included in the set of clustering markers due to their high heterogeneity of expression among samples. However, CX3CR1 and CD33 were added to the 23 clustering markers on heatmaps for the phenotypic characterization of myeloid cells.

### Phenotypic Characterization of Identified Cell Clusters

We used the SPADEVizR R-package to perform the phenotypic categorization of the 150 cell clusters (26). Expression level of each marker was classified into five categories, which were defined based on the range of expression (5th−95th percentile) relative to the myeloid population gate across all samples. The five uniform categories represent negative, low, medium, high, and very high levels of marker expression. The categories of expression were displayed using a color scale, which ranged from white to dark red. The dendrogram, represented in the heatmap, was constructed using Euclidian distance and the complete linkage method.

There were 36 clusters that displayed a lymphoid phenotype (based on the negative expression of CD33, CD123, CD14, and LILRB3), which were identified among the total 150 cell clusters and were removed from the SPADE analysis to leave only myeloid cell clusters. Additionally, 11 myeloid cell clusters with <350 cells associated were removed from the analysis, as their phenotypes cannot be assessed accurately. Thus, 103 myeloid cell clusters were remaining from the 150 SPADE clusters.

Families of cell clusters were determined using hierarchical clustering, represented by a dendrogram on the top of the marker categorical heatmap. Thanks to this clustering method, cell clusters with similar marker expression profiles were grouped together in the heatmap. The dendrogram was cut so that the different cluster families with homogeneous expression profiles were separated. This resulted in 12 different cluster families. These cluster families were also represented in a tSNE representation. The tSNE representation was generated using the original 23 clustering markers and Barnes-Hut t-Distributed Stochastic Neighbor Embedding (53), based on a perplexity parameter of 3. Distances between cell cluster phenotypes were calculated based on the Manhattan distance.

### Identification of Differentially Abundant Clusters and Correlating Clusters

We used the SPADEVizR R-package to identify differentially abundant clusters (DACs), and correlated clusters (CCs) (26). DACs identification was based on the percentage of cells in the clusters, relative to total myeloid cells by unpaired Student *t*-tests (absolute fold-change > 2 and *p* < 0.05). CCs identification was based on the percentage of cells in clusters relative to the total myeloid cells that correlated with HIV RNA levels (absolute Pearson correlation coefficient > 0.65 and *p* < 0.05).

### Quantification of Cluster #39 Phenotypic Specificity

Detailed phenotypic characterization of cluster #39 relative to CD1c^+^ cDC and CD32b^+^ CD1c^+^ cDC clusters was performed using CytoCompare R-package (54), based on the Kolmogorov-Smirnov distance, using a threshold of 0.30.

### Statistical Analysis of Cluster Abundances Among Conditions

A two-tailed unpaired Welch's *t*-test, with a *p*-value threshold of 0.05, was used to compare the percentages of cDC2 and pDC among CD45^+^ cells and the differences in cluster cell abundance between each biological condition. All statistical analyses were performed using GraphPad Prism 7.0 (GraphPad Software).

## Data Availability Statement

The normalized cytometry profiles were deposited on the FlowRepository database and are accessible via the accession number FR-FCM-ZYGJ.

## Ethics Statement

This study and the associated protocol were approved by the Ethics Committee of Cochin Hospital and ethics review committee of Ile de France VII. All subjects gave written informed consent in accordance with the Declaration of Helsinki.

## Author Contributions

SC, A-SB, OL, RL, and BF: conceptualization. SC, NT, LA, A-SB, OL, and BF: methodology. SC, NT, LA, BV, CB, CG, CL, PB, RL, A-SB, OL, and BF: validation. SC, NT, BV, A-SB, OL, and BF: formal analysis and writing—review and editing. SC, NT, and BF: investigation. CG, CL, PB, and A-SB: resources. SC, NT, and BF: writing—original draft. BF: supervision.

### Conflict of Interest

The authors declare that the research was conducted in the absence of any commercial or financial relationships that could be construed as a potential conflict of interest.

## Supplementary Material

Figure S1 Myeloid gating strategy. “Singlets” were identified using cell length vs. Ir191-DNA intercalator and calibration beads signal was excluded (cells no beads). Living leukocytes were identified by selecting Rhodium (Rh103)Di-negative cells, and then CD45^+^ cells. Finally, myeloid cells were identified by gating on CD3^−^, CD19^−^, and then NKp80^−^ HLA-DR^+^ cells.

Figure S2 Categorization of marker expressions. Marker expression ranges were used to characterize the phenotypes of identified SPADE clusters. Ranges of marker expression were obtained after manual gating of myeloid cells and are presented as arcsine transformed Mean Signal Intensity (MSI) signals. The range of expression for each marker was defined based on the 5th−95th percentiles of expression throughout the dataset, and were divided into five categories represented using a five-tiered color scale ranging from white (not expressed) to dark red (highly expressed). For each expressed marker of a particular cluster, the average of the median marker expression level was used to determine the expression category of that marker. Combined marker ranges define the phenotype of each cluster. Clustering markers are shown in blue.

Figure S3 tSNE representation showing the phenotypical similarities between cell clusters identified by SPADE. Each dot corresponds to a cell cluster and the dots are positioned in a 2-dimensional space that best represents the phenotypical proximity between cell clusters. Cell clusters have been colored based on their associated cell cluster family, blue for monocyte families, red for cDC families and green for pDC family.

Figure S4 Cell number in each myeloid SPADE cluster. This representation shows the number of cells associated with each myeloid cell cluster, regardless of sample cell origin. Cluster names are indicated on the X-axis and the corresponding number of cells on the Y-axis. The size of the dots is proportional to the number of cells in the cluster. Cell clusters are ordered based on the dendrogram represented in Figure 2.

Figure S5 Identification of differentially abundant clusters for each biological condition comparison. **(A–C)** Volcano plot representations showing Differentially Abundant Clusters (DACs) in HIV controllers, primary HIV and HIV cART samples compared to Healthy samples. **(D–F)** Volcano plot representations showing DACs in HIV controllers and primary HIV samples compared to HIV cART samples and HIV controllers compared to primary HIV samples. Each dot in the representation corresponds to a cell cluster and is proportional in size to the number of cell associated. Log_2_ fold-changes are indicated in the X-axis, and the associated *p*-values, shown as −log_10_, are indicated in the Y-axis. DACs were identified using a paired Student *t*-test with a *p*-value threshold of 0.05 and a fold-change cutoff of 2. Red dots correspond to significantly abundant clusters.

Figure S6 Marker expression densities showing the phenotypic specificity of cluster #39 relative to CD1c^+^ cDC clusters. The expression densities of all clustering markers, as well as CD33 and CX3CR1 are shown for cluster #39 and CD1c^+^ cDC clusters. The marker expression densities for cluster #39 are shown in blue whereas the marker expression densities for all CD1c^+^ cDC clusters are shown in gray. The differences between marker expression densities were quantified using the Kolmogorov–Smirnov distances (KS) using the entire and singular dataset.

Figure S7 Marker expression densities showing the phenotypic specificity of cluster #39 relative to CD32b^+^ CD1c^+^ cDC clusters. The expression densities of all clustering markers, CD33 and CX3CR1 are shown for cluster #39 and CD32b^+^ CD1c^+^ cDC clusters. The marker expression densities for cluster #39 are shown in blue whereas the marker expression densities for all CD32b^+^ CD1c^+^ cDC clusters are shown in gray. The differences between marker expression densities were quantified using the Kolmogorov–Smirnov distances (KS) using the entire and singular dataset.

Table S1 Overview of the mass cytometry panel. The metal, antigen, clone, isotype, and supplier are indicated for each antibody. ^*^Not used for cell clustering.
